# Identifying *thriving* Workplaces in Hospitals: Work Characteristics and the Applicability of Character Strengths at Work

**DOI:** 10.1007/s11482-018-9693-1

**Published:** 2019-01-24

**Authors:** Cornelia Strecker, Alexandra Huber, Thomas Höge, Melanie Hausler, Stefan Höfer

**Affiliations:** 1 Institute of Psychology, University of Innsbruck, Innrain 52, 6020 Innsbruck, Austria; 2 Department of Medical Psychology, Medical University of Innsbruck, Innsbruck, Austria

**Keywords:** Work characteristics, Autonomy, Applicability of signature character strengths, Work engagement, Well-being

## Abstract

In most of their work settings, the health and well-being of hospital physicians are at risk. Trends of work intensification and changing laws in the European Union and beyond have heightened the call for taking a closer look at the workplace and training conditions of hospital physicians. This study aims to identify specific work characteristics (such as autonomy, social support, cognitive demands, and skill adequacy), in order to determine conditions for the applicability of individual character strengths at work and in turn for increased work engagement and well-being. We examined our hypotheses based on cross-sectional (*N* = 173) and longitudinal self-report data (*N* = 72) of hospital physicians in Austria. The results identified significant indirect effects of skill adequacy, cognitive demands, autonomy, and social support at work – via the applicability of individual character strengths at work – on work engagement and general well-being. Longitudinal analyses additionally confirmed autonomy as a *thriving work characteristic* for promoting the applicability of individual character strengths over time (time lag: 6 months). This study revealed the value of enabling and preserving the applicability of character strengths in a hospital work setting and focused – for the first time – on its predicting work characteristics. Furthermore, it emphasizes the importance of securing skill adequacy early in the training of young physicians and encouraging, as well as, sustaining autonomy in their daily work life.

## Introduction

The prevalence of burnout among physicians (e.g., Shanafelt et al. 2015), recent developments in the implementation of EU’s health and safety laws (European Commission 2003), and a general trend of work intensification (Richter et al. 2014) have made it imperative to take a closer look at the workplace and training conditions of hospital physicians in order to enhance their health and well-being. This investigation does not only potentially benefit the physicians, but also their patients and beyond that, the health care system of a society in general.

Theoretical assumptions in the field of Positive Psychology (Peterson and Seligman 2004) and research results (e.g., Harzer and Ruch 2013; Hausler et al. 2017a) point to strong relations between the application of individual character strengths and both general and work related well-being. Our approach was to investigate factors that enable and safeguard the ability of hospital physicians to apply their individual character strengths as personal resources at work. To the best of our knowledge, these predictors have not yet been systematically studied. This study fills a gap, in identifying those work characteristics that determine conditions for the applicability of character strengths in hospital settings. We focused on four work characteristics, which – according to earlier theories – could function as ideal work conditions: autonomy and social support (typical work resources), cognitive demands (for learning at work) and skill adequacy (qualification; person-organization-fit). In sum, we investigated the relationship between these specific work characteristics, the applicability of individual character strengths at work and work engagement as well as well-being.

This interdisciplinary study addresses the promotion of well-being, health, personal development, and a “good life” at work or in general, as they are covered in the following three sub-disciplines: (i) Health Psychology, the study of “[…] social and psychological factors” (APA 2017) influencing health and illness and additionally aiming to improve the health care system. (ii) Work and Organizational Psychology, the study of human workplaces that promote worker health and foster well-being, performance, and personal development. (iii) Positive Psychology, the “study what is good in life and why it is worth living” (p. 290, Vázquez and Chaves 2016), with a focus on positive subjective experiences (e.g., well-being or vitality), positive individual characteristics (e.g., character strengths or talents), positive interpersonal relationships (e.g., appreciation or trust) and positive institutions (e.g., families or organizations) (Seligman and Csikszentmihalyi 2000; Peterson and Seligman 2004).

## Research Background

### General Work Situation and Well-Being of Hospital Physicians

The work situations of many hospital physicians appears to be rather unpleasant. For Montgomery (2014) physician burnout is “inevitable” (p. 50) in healthcare organizations, among other things, due to contradictory conditions. Research done in the 1980s in the US showed that burnout is in particular an issue of human service professions, like nurses or physicians (Leiter and Maslach 1988). Scandinavian and British studies in the 1990s showed, according to the *British Medical Association*, that “there are claims of stress and `burnout´ among physicians and data demonstrate increased risk of clinical depression and suicide” (Aasland et al. 1997); these studies also showed a prevalence of psychiatric morbidity of 27% in physicians (Ramirez et al. 1996). A 2012 U.S.-based study (Shanafelt et al.) demonstrated that, out of 7288 physicians, almost half (46%) reported at least one symptom of burnout, which three years later increased to 54% (Shanafelt et al. 2015). Shanafelt and colleagues also argued, in 2012, that, compared to the general population, physicians in the U.S. were more likely to have symptoms of burnout (37.9% vs. 27.8%) and to be dissatisfied with their work-life balance (40.2% vs. 23.2%). These results were confirmed and even intensified in their report of 2015. The peak of risk seemed to occur in the first years of specialty training in hospitals (Shanafelt et al. 2015).

In Germany and Austria, researchers have found a similar prevalence of burnout (up to 50%) and depression (10%) in physicians (Weigl et al. 2012; Wurm et al. 2016).

Studies have primarily pointed to critical job strains as possible causes: lack of autonomy, inadequate resources and social conflicts as well as organizational stressors, workload, and adverse working hours (e.g., Angerer and Weigl 2015; Lee et al. 2013; Linzer et al. 2009; Geiger-Brown and Lipscomb 2010).

Work conditions and legislation related to work pose an additional challenge for a majority of hospital physicians in Europe, particularly the application of the *European Working Time Directive* (European Commission) of 2003, which protectively sets weekly work hours at 48 per week on average. These conditions should have led to the demand for new recruitment, as the workload is the same but physicians have less working time. Instead, they led to work intensification for the present staff in most cases (Germany: Richter et al. 2014) and reduced availability of medical staff (UK: Fitzgerald and Caesar 2012). A recent German study in a paediatric university hospital identified the additional burden of a proportion of less than one third (31.2%) of physicians` time “dedicated to direct patient contact” (transl., p.21, Heinen et al. 2016). International competition fostering the development of new complex technologies and cost-cutting measures has reinforced this development for many years (Kumar 2011).

Furthermore, profession-inherent demands such as emotional workload need to be considered as well. Due to regular patient contact, emotional regulation is always demanded from physicians. However, compared to the current working conditions mentioned above, profession-inherent demands have even less potential to be modified, even if framework conditions change.

The professional situation of hospital physicians is characterized by a generally higher risk of burnout as well as mental health challenges, most probably due to a long-standing work situation accompanied by strains like work intensification, high (emotional / quantitative) workload, and a demotivating level of administration during the workday. On the other hand, physicians are still considered to have a high probability of meaningful experiences at work, personal fulfilment and recognition, due to the potential positive impact of their work on other people’s lives. Ramirez and colleagues (Ramirez et al. 1996) recommended “maintaining or enhancing job satisfaction” (p. 724) by protecting consultants’ mental health against the high demands of medical practice.

Thus, we concluded that there is a need to investigate what enables hospital physicians to pursue their vocation and preserve their personal resources at work in a positive, non-detrimental way.

### The Applicability of Signature Character Strengths at Work (ASCS-W)

Peterson and Park (2006) suggest that character strengths are important resources in a work context because they positively influence people’s behavior. Several studies have found positive relations between the possession of certain character strengths and positive work-related outcomes like job satisfaction, well-being (e.g., Littman-Ovadia and Steger 2010), job performance (e.g., Harzer and Ruch 2014) and coping with work-related stress (e.g., Harzer and Ruch 2015). One study analyzed relations between the endorsement of character strength and the burnout of practitioners (Steffanina 2015). The study showed the importance of fostering the application of character strengths in the work context of healthcare professionals. Where numerous studies connect the possession of character strengths and work-related outcomes, Littman-Ovadia and Steger (2010) argued that the ability to use character strengths in the workplace is even more important for job satisfaction. In line with this argument, specific strength *applicability* questionnaires were developed (e.g., Harzer and Ruch 2013; Littman-Ovadia and Steger 2010). The *Applicability of Character Strengths Rating Scales* (ACS-RS) by Harzer and Ruch (2013), for example, considers the impact of fostering or hindering situational and individual circumstances on individual behavior relevant to the respective character strength. The scales refer to *possibilities* in the work context that are generated by the individual, other people or work tasks.

The applicability of character strengths *at work* is associated with a range of positive outcomes such as life satisfaction (Allan and Duffy 2014; Douglass and Duffy 2014; Harzer and Ruch 2016), different aspects of well-being (e.g., pleasure, engagement, and meaning; Harzer and Ruch 2013; Littman-Ovadia and Steger 2010), work-related outcomes like job satisfaction (Harzer and Ruch 2013; Lavy and Littman-Ovadia 2017; Littman-Ovadia and Steger 2010), employee engagement (Crabb 2011; Lavy and Littman-Ovadia 2017), work productivity and organizational citizenship behavior (Lavy and Littman-Ovadia 2017). Although we know quite a lot about the outcomes, there has not been much research on *predictors* of the applicability of character strengths. Also, the use or applicability of character strengths in special working contexts, such as in health care, has not been examined before. However, a recent study reported positive relations between the applicability of character strengths and well-being / health in samples of medical students and resident physicians (Hausler et al. 2017a). The authors emphasized the great potential of the applicability of character strengths as a conditional resource in the working context.

Harzer and Ruch (2013) reported that the more relevant a strength was for an individual, the higher the increase of positive experiences. They concluded that character strengths, in general, are relevant in the vocational context, independent of their specific content. On the other hand, other studies supported the importance of specific character strengths. For example, the character strength of perseverance was found to correlate most highly and positively with work performance and most negatively with counterproductive work behaviors (Littman-Ovadia and Lavy 2016). More research could be done regarding correlates of specific character strengths.

Lavy and Littman-Ovadia (2017) suggest the *broaden-and-build theory* (Fredrickson 2001) as a possible underlying mechanism for the positive effect of the ultimate *application* of character strengths on work-related outcomes. Like in an upward spiral, the theory assumes that “positive emotions broaden people’s momentary thought-action repertoires, which in turn, serves to build their enduring personal resources” (Fredrickson 2001, p.218). In this context, they suggest positive affect and work engagement as possible mediators for the link between the application of character strengths and work-related outcomes (e.g., job satisfaction and productivity) that could be confirmed in their study. Another possible mechanism of positive effects could be the awareness of self-efficacy (Bandura 1977), which is an important term in the context of individual development and clearly associated with a wide range of positive work-related outcomes (e.g., Stajkovic and Luthans 1998; Judge and Bono 2001).

When considering the work situations of hospital physicians, several researchers focus on the topic of improving the well-being of healthcare professionals. However, little evidence exists as to how to address this problem effectively (Shanafelt et al. 2012). Also, conducted interventions have primarily focused on individual strategies to address the problem and were not evaluated in a formal way (Knight et al. 2017). In their work, Dyrbye and colleagues (Dyrbye et al. 2014) therefore claimed the need for *organizational* interventions “to address the curricular, training, and system factors that also contribute to the high prevalence of distress” (p. 449) among physicians. In addition, although there is evidence about health-promoting working conditions in the health care sector, we do not know how to foster physicians’ use of their character strengths.

One study began to look at work characteristics and the outcome of employees` fulfillment of their potential at work: Lavy et al. (2017) recently showed, in a ten-day diary study that the resource “supervisor support on a given day” led to “increased strengths use on the following day” (p. 703). More systematic research is needed to identify condition-related predictors.

To this end, we focused on work characteristics that may foster hospital physicians` applicability of character strengths at work, leading to positive experiences and allow individuals to thrive (upward spirals). The focus on enhancing the opportunity to use one’s character strengths is a new and potentially very effective way to safeguard well-being of physicians.

Throughout this paper the well-being term *thriving* will refer to Su, Tay and Diener’s definition (2014), where thriving is a “state of positive functioning at its fullest range - mentally, physically and socially” (p. 256) including subjective as well as psychological well-being. In accordance with that definition, *thriving work characteristics* refer to working conditions that foster an individual’s mental, physical and social states of positive functioning, directly linked to health, well-being, engagement, and personal growth.

### Work Characteristics that Allow People to Thrive: Theories of Demands, Resources and Stressors at Work

Several models and theories of Work and Organizational Psychology contain elements which correspond to the afore mentioned concept of *thriving work characteristics*. Further to the previous section of this paper, the use of individual character strengths can firstly be seen as essential part of personal growth and, secondly – as described above – strongly associated with different aspects of well-being / thriving (positive emotions, engagement, satisfaction). Therefore, we argue that work characteristics that conceptually have been shown as personality-promoting work characteristics, also have the potential to foster the applicability of individual character strengths at work. To reiterate and further specify the definition, *thriving work characteristics* should have the capacity to affect the applicability of character strengths at work. In the following, we describe which characteristics may theoretically be suited best to the concept of *thriving work characteristics*.

The *Job Demands-Resources model* (JD-R; Bakker and Demerouti 2007) distinguishes between demands and resources at work. Demands, as they describe them, are “associated with certain physiological and/or psychological costs” (Bakker and Demerouti 2007, p. 312) and are conceptualized as potential stressors. By contrast, resources are able to increase motivation (e.g., work engagement) as they are “functional in achieving work goals [,] […] stimulate personal growth, learning, and development” (Bakker and Demerouti 2007, p. 312). Subsequently, resources may lead to better health as they can alleviate the costs of health-impairing demands. Social support and autonomy/job control have become known as two central job resources (Semmer and Udris 2004). Both include and/or promote (decision) latitudes, which enable possibilities for individually preferred behavior (like applying individual character strengths). When employees have the possibility to use own ideas in performing the work tasks or to decide for themselves what tasks to pursue (autonomy), the probability of applying individual preferences and strengths at work strongly increases. A similar effect is to be expected when employees feel safe to unfold personal preferences/strengths due to a supportive, non-condemning work environment (social support). Thus, the resources autonomy and social support comply with the concept of *thriving work characteristics*.

Since empirical results (e.g., Cavanaugh et al. 2000) have shown that job demands (cf. JD-R) do not always impair performance and job satisfaction, LePine et al. (2005) classified stressors into *challenge* and *hindrance* stressors. According to their definition, challenge stressors are able to activate learning and personal growth, whereas hindrance stressors threaten regulation capacities and health. Karasek (1979) described in his Job strain model (later Demand-Control Model) that high job demands – combined with a high job decision latitude – characterize an active job meaning motivation and active learning. These definitions suggest that challenge stressors/demands basically have the potential to be categorized as *thriving work characteristics*. Furthermore, Glaser et al. (2015) proposed an *Integrated Model of Learning Demands, Resources and Stressors*, which integrates different established approaches and theories (including the above), mainly based on the *Action Regulation Theory* (Frese and Zapf 1994). In their model, the learning demands (e.g., cognitive demands) and the resources (e.g., social support or autonomy) foster positive outcomes like well-being, engagement, and performance – successively in terms of *personality development*. Learning demands thereby correspond more or less to challenge stressors (LePine et al. 2005) and are therefore not necessarily associated with negative outcomes but rather activate personal development. In keeping with the *Action Regulation Theory*, learning demands by comparison represent a more objective approach in defining person-independent work characteristics. Glaser et al. (2015) confirmed the proposed relations empirically, identifying positive effects of cognitive demands, autonomy, and supervisor feedback on intrinsic motivation and creativity. Resources (autonomy and supervisor feedback) additionally led to a decrease in emotional irritation as one aspect of health impairment. Work characteristics pertaining to learning demands and resources in this model (Glaser et al. 2015) can therefore also be seen as *thriving work characteristics*. Especially *cognitive demands* relate to challenge stressors (LePine et al. 2005), to an active job (Karasek 1979) and to the model and findings from Glaser et al. (2015) regarding learning demands, as well as they can be applied to the profession of highly educated physicians. A work environment with cognitive demands typically enables learning, personal growth and in order to meet the challenge successfully, the chance to apply individual character strengths. Thus, we argue that cognitive demands have the capacity to be a *thriving work characteristic*.

In order to generate positive work experiences (e.g., individual work attitudes, no stress, prosocial behavior or performance) the P-O fit model (Kristof 1996) indicates the precondition of a need-supplies and a demands-abilities related fit at work. Harzer and Ruch (2013) introduced the argument that a “congruence between the job tasks and the individual signature strengths can be interpreted as both a need-supplies and demands-abilities related fit” (p. 979) referring to the P-O fit concept of Kristof (1996). *Signature character strengths* are thereby defined as (about three to seven) central strengths which are typical of an individual; they are highly distinctive and associated with passion, authenticity, learning, desire, and power (Peterson and Seligman 2004). Moreover, the literature emphasizes available opportunities to use one’s capacities for facilitating job satisfaction, engagement, or productivity at work (e.g., Lowe 2010). Another relevant part of *thriving work characteristics* – in terms of personal and situational *prerequisites* for thriving at work – is a general fit between the person and the job. This fit differs from individual growth and the chance of applying personal character strengths at work (see ACS-RS); for it refers to a basic qualification for the job, an adequacy of personal skills (such as theoretical knowledge and practical skills) that are important for every (in this case) physician to have. Personal skill adequacy should facilitate the chance of applying individual character strengths at work as the employee is in her/his “right” job. Thus, we argue that skill adequacy has the capacity to be a *thriving work characteristic*.

The universal importance of *appropriate* work characteristics in the field of hospital physicians was mentioned by Schneider et al. (2014). The authors demonstrated significant connections between work characteristics in hospitals with young physicians` burnout and engagement and concluded: “Attempts to strengthen well-being are advised to foster doctors’ capabilities to manage workload efficiently and to promote their abilities to gain resources for successful coping” (p. 1).

According to these presented models and theories, our study investigated *thriving work characteristics* as predictors of the ASCS-W and focused on two subsequent positive outcomes: Work engagement as work-related indicator and general well-being as universal indicator of positive functioning and successful coping.

## Hypotheses

According to the conceptualization of resources within the JD-R model, the concept of cognitive demands as one form of positive learning demands within the integrated model by Glaser et al. (2015), and the P-O fit model stressing a fit between abilities and demands, we propose autonomy, social support, cognitive demands, and skill adequacy as *thriving work characteristics*.

Hypothesis 1: Autonomy, social support, cognitive demands, and skill adequacy at work relate positively to the ASCS-W.

Based on research in the field of Positive Psychology (e.g., Harzer and Ruch 2013), we additionally propose that the applicability of character strengths in a work context affects a broad range of desirable outcomes and better functioning at work like work engagement and general well-being.

Hypothesis 2a: The ASCS-W relates positively to work engagement.

Hypothesis 2b: The ASCS-W relates positively to general well-being.

**Fig. 1:**
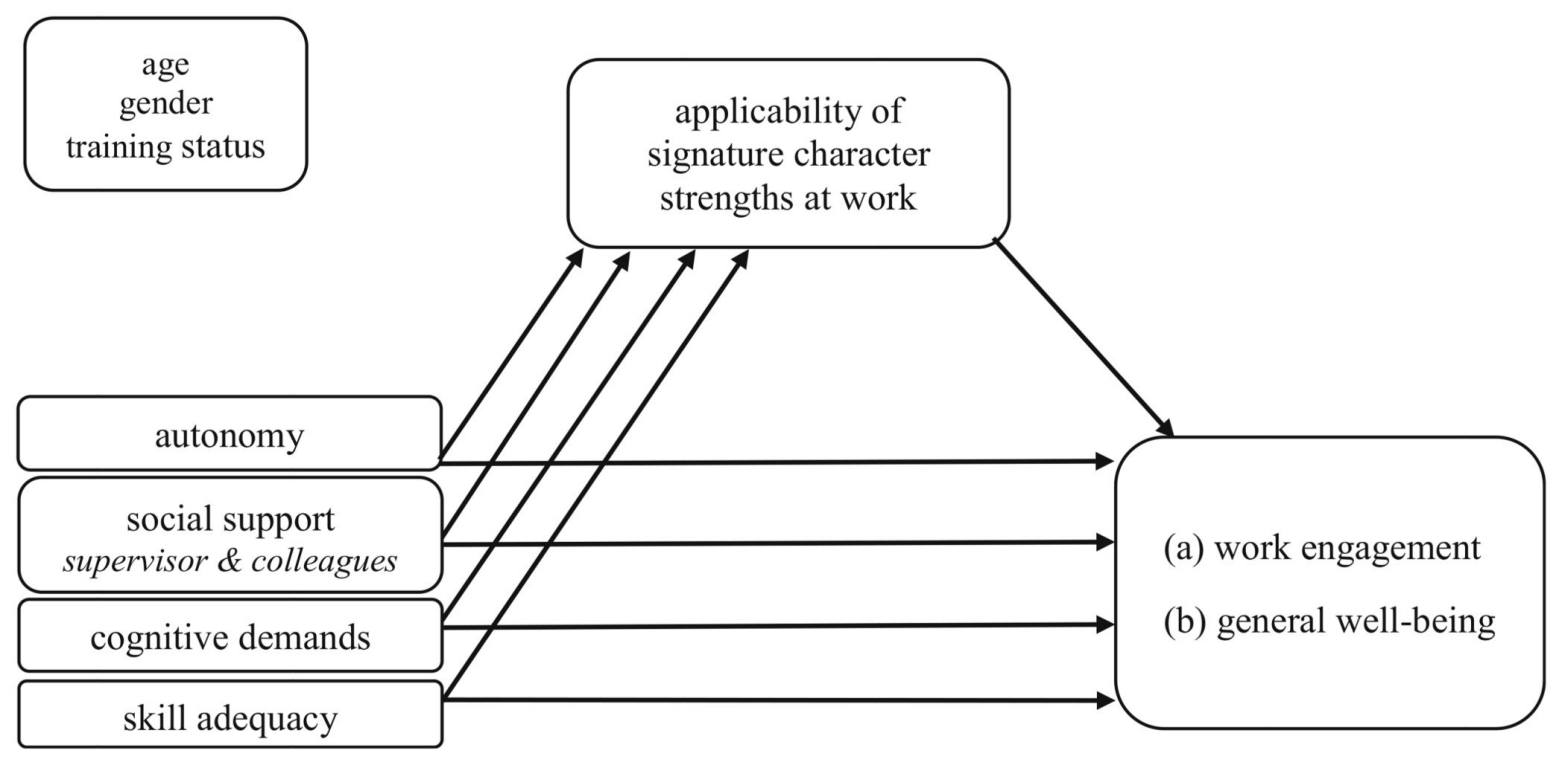
Hypothesis 3a & b: Indirect Effects through ASCS-W; controlled for age, gender and training status

Work characteristics which enable the ASCS-W may, firstly, establish an improved congruence and P-O fit (Kristof 1996) with corresponding outcomes and, secondly, lead to perceived self-efficacy (Bandura 1977). Like in an upward spiral, these mechanisms further lead to an improvement of positive work-related outcomes like work engagement and general well-being. We therefore attribute an indirect effect between *thriving work characteristics* and work engagement as well as general well-being, respectively, via the ASCS-W (Fig. 1):

Hypothesis 3a: Thriving work characteristics (autonomy, social support, cognitive demands, skill adequacy) have an indirect effect on work engagement through the ASCS-W.

Hypothesis 3b: Thriving work characteristics (autonomy, social support, cognitive demands, skill adequacy) have an indirect effect on general well-being through the ASCS-W.

To our knowledge, specific work characteristics as predictors of the ASCS-W have not been systematically analyzed before. We were interested in the effect of work characteristics as predictors of the ASCS-W *over time* to address potential causality effects:

Hypothesis 4: Thriving work characteristics positively affect the ASCS-Wover time (6 months):

Hypothesis 4a: Autonomy at work positively affects the ASCS-W over time (6 months).

Hypothesis 4b: Social support at work positively affects the ASCS-W over time (6 months).

Hypothesis 4c: Cognitive demands at work positively affect the ASCS-W over time (6 months).

Hypothesis 4d: Skill adequacy at work positively affects the ASCS-W over time (6 months).

## Methods

### Participants and Procedure

The cross-sectional sample consisted of *N* = 173 German-speaking hospital physicians of two hospitals in Austria (response-rate: 20.1%). The physicians (65% women, 35% men) came from various medical disciplines (e.g. anesthesia, internal medicine, psychiatry, radiology, and trauma surgery). The mean age of the total sample was 33.4 years (*SD* = 6.7, range = 24–64 years). 154 physicians indicated to be in training and 20 to be medical specialists. Most of the physicians (81%) worked at a big university hospital, 9% at a smaller hospital.

We invited the physicians at the two hospitals via e-mail to participate in the online survey contained within a larger research project, with the prospect of rewards (e.g. raffle of brunch vouchers for two). After a time period of 6 months all participants from T1 were invited to participate in a follow-up (T2). A total of *N* = 72 physicians took part at T2. The longitudinal sample did hardly differ from the cross-sectional sample. Gender (65% women, 35% men) and age (mean = 32.9 years, *SD* = 5.2, range = 25–51 years) were comparable, the majority indicated to be in training (*N* = 63), 8% to work at the smaller hospital and the disciplines mentioned above were again represented most often. With regard to the participants who did not complete T2, we noticed more missing values in general, but an equivalent picture regarding demographics and the specification of indicated work characteristics and outcomes. Drop out reasons might therefore be associated with methodical / job-specific aspects such as the high time requirement for completing the survey, usual overstimulation of information by e-mail, or general high workload in this profession. The latter points are likely to apply as reasons for the general response rate at T1, too.

### Measures

All following measures were applied at T1 and T2, respectively:

#### Thriving Work Characteristics

The work characteristics autonomy (9 items; ‘I am free to determine how I do my work’), cognitive demands (4 items; e.g., ‘My work requires me to continually weigh various topics and to set priorities before I can get things done’) and skill adequacy (4 items; e.g., ‘My theoretical skills correspond with the work demands of my field’) were selected from the self-report version of the *Activity and Work Analysis in Hospitals* (TAA-KH-S; Büssing and Glaser 2002) in an adapted screening version. The measure distinguishes between challenging work demands, work resources, and work stressors corresponding to the model of Glaser et al. (2015). The response format on a five-point Likert scale ranges from *no, not at all* (=1) to *yes, definitely* (=5). Cronbach’s alpha (*α*) for *skill adequacy* revealed in this study .72, for *cognitive demands* .77 and for *autonomy* .90.

Social support by co-workers (3 items) and supervisors (3 items) was measured with the subscales for social support from the *Salutogenic Subjective Work Analysis* (SALSA; Rimann and Udris 1997). Each item (e.g., ‘How much can you rely on [your colleagues/supervisors] if you have problems at work?’) was rated on a five-point scale ranging from *not at all* (=1) to *absolutely* (=5). The arithmetic combination of colleagues and supervisors substantially represents the perceived social support *at work* and can be justified in the sense of a formative construct (Diamantopoulos and Siguaw 2006). Cronbach’s alpha for *social support at work* (colleagues and supervisors) was .87 in this setting.

#### Applicability of Signature Character Strengths at Work

For measuring ASCS-W, we first used the 120-item version of the *Values in Action Inventory of Strengths* (VIA; Höfer et al. 2018 in this special issue; Littman-Ovadia 2015; original: VIA Institute on Character 2014) to measure the top five character strengths. This version is used by the VIA Institute on Character (2014) as standard questionnaire. Psychometric properties were similar to the original 240 items version with Cronbach’s alpha in this sample ranging from .61 (teamwork) to .90 (spirituality). Item examples are: ‘I always keep my promises’ (authenticity); ‘I am never too busy to help a friend’ (kindness); ‘I am always willing to take risks to establish a relationship’ (love). The five-point scale ranges from *strong disagreement* (=1) to *strong agreement* (=5).

In addition, we used the *Applicability of Character Strengths Rating Scales* (ACS-RS; Harzer and Ruch 2013) to evaluate the applicability of the top five signature character strengths in the work context (ASCS-W). For each strength, four items (concerning demand, helpfulness, personal relevance and actual behavior) had to be rated on a five-point Likert scale from *never* (=1) to *(quite) always* (=5). To analyze the applicability of the signature character strengths at work, we computed the mean value of the applicability of each of the top five strengths. Internal consistency in the present study of the ACS-RS was *α* = .80.

This two-step procedure was developed and validated by Harzer and Ruch (2013).

#### Work Engagement

Work engagement was measured with the German nine-item short-version of the *Utrecht Work Engagement Scale* (UWES; Schaufeli and Bakker 2003; Schaufeli et al. 2006). The response format on a seven-point Likert scale ranges from *never* (=0) to *always* (=6). An item example is: ‘At my job, I feel strong and vigorous’. Cronbach’s alpha internationally varies between .85 and .92 (Schaufeli et al. 2006), in this study it was .94.

#### General Well-Being

The *Comprehensive Inventory of Thriving* (CIT; Hausler et al. 2017; Su et al. 2014) was used to measure general well-being and comprises 54 items. The measure consists of seven different components: (1) subjective well-being, (2) relationships, (3) engagement, (4) meaning in life, (5) accomplishment, (6) autonomy, and (7) optimism, which are measured on a five-point scale from *I strongly disagree* (=1) to *I strongly agree* (=5) (Su et al. 2014). The seven components can be further categorized into the two main-components of well-being: subjective and psychological well-being. Item examples are: ‘My life is going well’ (subjective well-being); ‘There are people I can depend on to help me’ (relationships / psychological well-being). The German version showed a very good Cronbach’s alpha of .96 (Hausler et al. 2017), in this study it was .89. As the latter highly intercorrelate (*r* = .91; Hausler et al. 2017, p. 224) and the focus of this paper lies on the work characteristics, we used the equally recommended total score of general well-being.

### Statistical Analyses

Hypotheses 1–3 were analyzed with the cross-sectional data (*N* = 173); Hypothesis 4 was analyzed with the longitudinal data (*N* = 72). Analyses were based on scale mean-scores concerning the following variables: *skill adequacy*, *cognitive demands*, *autonomy*, *social support at work*, the applicability of signature strengths at work, work engagement, and general well-being. No outliers above or below a *z-*value of ± 3.29 were identified.

Demographic analyses and correlations were done with SPSS Version 24. Analyses of indirect effects were performed using the SPSS PROCESS macro Release 2.16.2 (Hayes 2013) using standardized z-values of the variables and applying bootstrapping-procedure (*N* = 5.000 bootstrap-samples) for computing confidence intervals for the indirect effects. The tests of the indirect effects regarding each work characteristic (predictors) in the PROCESS macro were always controlled for the three other work characteristics, in order to allow presenting all of them in one result model.

Additionally, all cross-sectional analyses were controlled for gender (1 = female, 2 = male), training status (1 = in training, 2 = specialist) and age (years) as continuous control-variable. Reasons for controls are the different group sizes of gender and training status, expected significant correlations of age and training status with some work characteristics and the explorative character of investigating predictors of ASCS-W.

Longitudinal analyses (cross-lagged panel) were performed through path analyses with Amos Graphics 24.

## Results

Descriptive statistics, psychometric properties and the inter-correlations for all scales are provided in Table 1. The arithmetic mean of *autonomy* was comparatively low (T1: *M* = 2.55), whereas the mean of *cognitive demands* (T1: *M* = 4.22) and the ASCS-W (T1: *M* = 3.90) were comparatively high in this sample.

Pearson’s coefficient inter-correlations in Table 1 can be interpreted as follows: *r* < .10 = no correlation, *r* = .10–.29 = low correlation, *r* = .30–.49 = moderate correlation, *r* ≥ .50 = high correlation (Cohen 1988). Cronbach’s alpha indicates acceptable internal consistency when values are >.70 (Nunnally 1978; see Peterson 1994). Only the variable *skill adequacy* at T2 did not meet this standard perfectly (*α* = .64). Because this scale is assessing the adequacy of theoretical, social and practical skills and therefore being more formative than reflexive (Diamantopoulos and Siguaw 2006), the lower internal consistency can be justified.

### Cross-Sectional Hypotheses 1–3

**Fig. 2:**
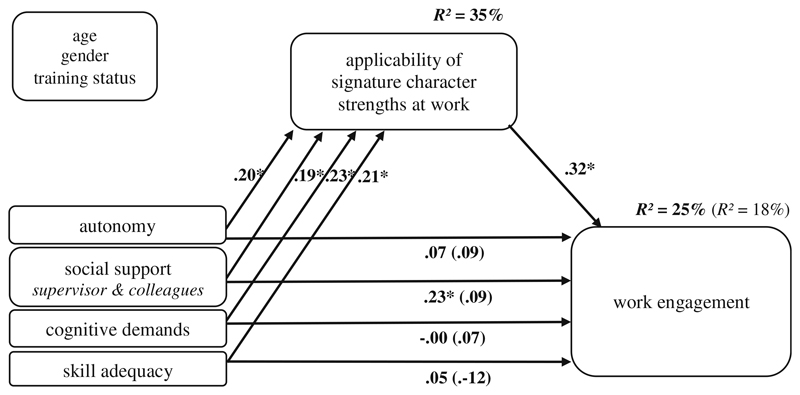
Model coefficients: work characteristics - ASCS-W - work engagement (*N* = 165); standardized coefficients; controlled for age, gender and training status; * *p* ≤ .05 (two-tailed)

**Fig. 3:**
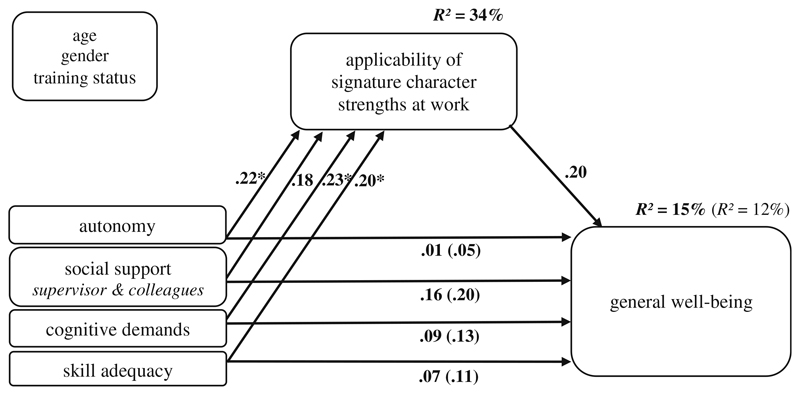
Model coefficients: work characteristics - ASCS-W – general well-being (*N* = 160); standardized coefficients; controlled for age, gender and training status; * *p* ≤ .05 (two-tailed)

The indirect-effect model (see Fig. 1) postulates work characteristics as independent variables and (a) work engagement as well as (b) general well-being as outcomes. The indirect effect between these relations should go through the ASCS-W. Analyses of T1 data show the following:

All four work characteristics revealed significant standardized regression coefficients between .19 and .23 on the ASCS-W in model (a) (Table 2, Fig. 2), confirming Hypothesis 1. Those effects resulted similarly in model (b), but the coefficient from the work characteristic *social support at work* on the ASCS-W (.18) was not significant (*p* = .053) (Table 3, Fig. 3). The control variables had no significant effects on ASCS-W and the outcomes.

#### Work Engagement

The correlation between ASCS-W and work engagement was positive and significant (*r* = .43, *p* ≤ .01, Table 1), confirming Hypothesis 2a. In addition, the direct effect of ASCS-W on work engagement in the model (controlled for work characteristics, Fig. 2) was positive and significant, too (β = .32, *p* ≤ .01).

Regarding Hypothesis 3a, the bootstrapping confidence intervals of the indirect effects supported the hypothesis of all four work characteristics indirectly affecting work engagement through the ASCS-W. The indirect effects via the four work characteristics varied between .06 and .07 (Table 2). Only the variable *social support at work additionally* had a direct impact (.23, *p* ≤ .05) on work engagement (Fig. 2). For bootstrapping confidence intervals and coefficients, see Table 2.

#### General Well-Being

The correlation between ASCS-W and general well-being was positive and significant (*r* = .31, *p* ≤ .01, Table 1), confirming Hypothesis 2b. In addition, the direct effect of the ASCS-W on general well-being in the model (controlled for work characteristics, Fig. 3) was positive (β = .20). However, the effect was not significant (*p* = .07; Table 3).

Regarding the indirect effects of Hypothesis 3b, we found significant indirect effects for the work characteristics *autonomy*, *social support* and *cognitive demands* on general well-being via the ASCS-W. The effects were smaller – compared to work engagement – varying between .04 and .05 (Table 3). Concerning *skill adequacy*, we did not find a significant indirect effect, as the confidence interval included 0 (Table 3). The bootstrapping confidence intervals of the other three indirect effects supported the hypothesis that the proposed work characteristics affect general well-being indirectly via ASCS-W. As we tested the hypothesized model with PROCESS, it is recommended to interpret only the indirect effects (and not the direct effects, see Hayes 2013). Thus, the results indicate a confirmation of Hypothesis 3b for *autonomy*, *social support* and *cognitive demands*, but not for *skill adequacy*.

#### Hypothesis 4: Work characteristics positively affect the ASCS-W over time

In order to take a closer look at the relation between work characteristics and ASCS-W, we performed cross-lagged-panel analyses with longitudinal data (Hypothesis 4).

**Fig. 4:**
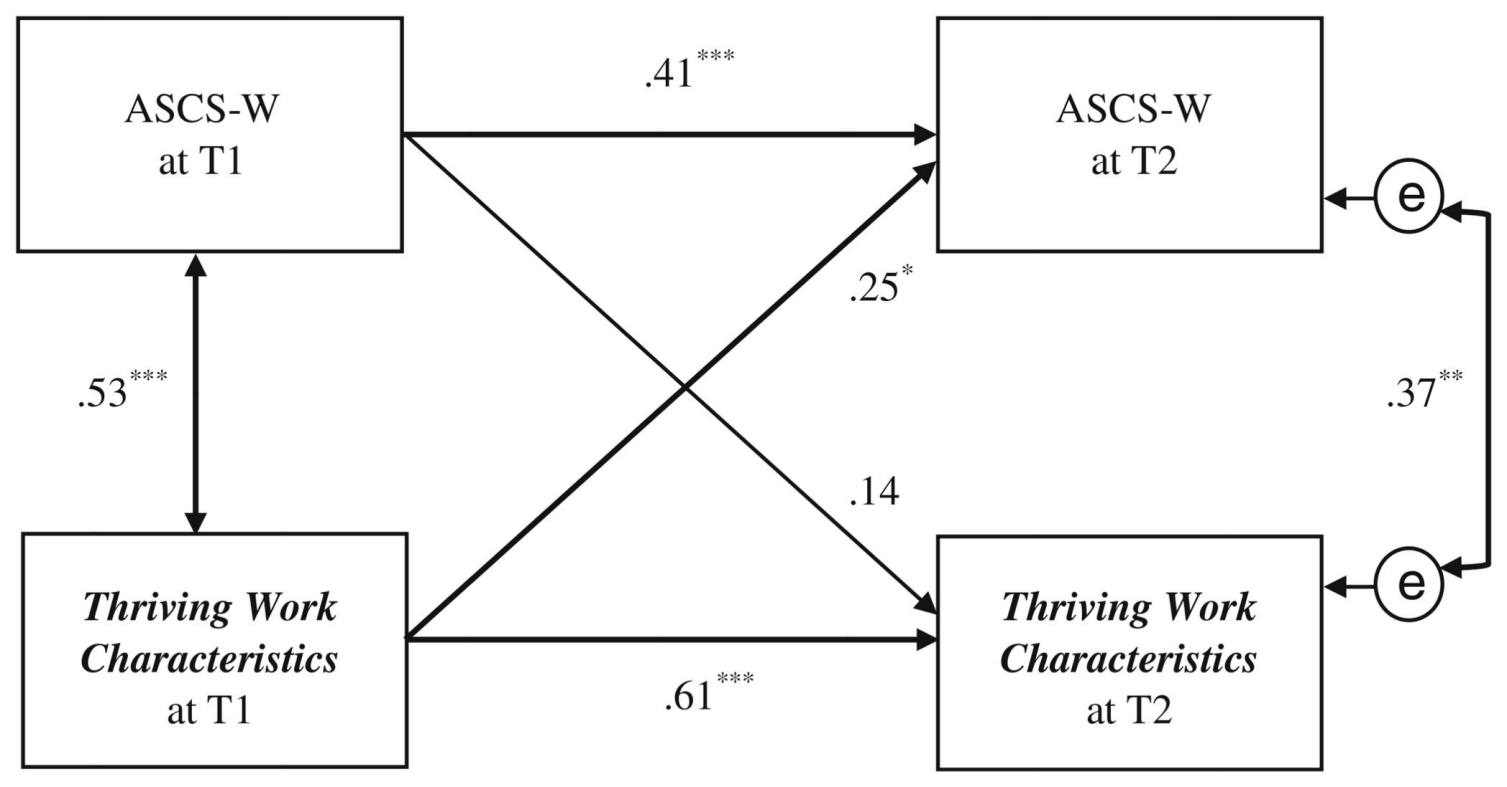
Path model of cross-lagged effects between the mean score of the four *thriving work characteristics* (autonomy, social support at work, cognitive demands and skill adequacy) and the ASCS-W at T1/T2 (*N* = 72); * *p* ≤ .05, ** *p* ≤ .01, *** *p* ≤ .001

We specified four direct paths from *thriving work characteristics* (T1) and the ASCS-W (T1) on *thriving work characteristics* (T2) and ASCS-W (T2) in a cross-lagged-panel model (Fig. 4). The manifest variables of the model represent the means of the four work characteristic scales at T1 / T2 and the means of the applicability of the individual top-five signature strengths at T1 / T2, respectively. The error terms of the dependent variables were allowed to correlate, reflecting that an assumed connection between work characteristics and ASCS-W at T2 is not only caused by the independent variables but other aspects not part of the tested model.

The coefficient of interest between the *thriving work characteristics* at T1 on the ASCS-W at T2 revealed a significant positive effect (β = .25, *p* ≤ .05) (Fig. 4), supporting Hypothesis 4. The reverse effect between ASCS-W T1 and *thriving work characteristics* T2 was not significant (β = .14, *p* = .18), confirming the hypothesized direction.

To gain a better understanding of effective and potential significant directions, we subsequently analyzed the model in more detail regarding each of the four work characteristics separately (Hypothesis 4a-d).

**Fig. 5:**
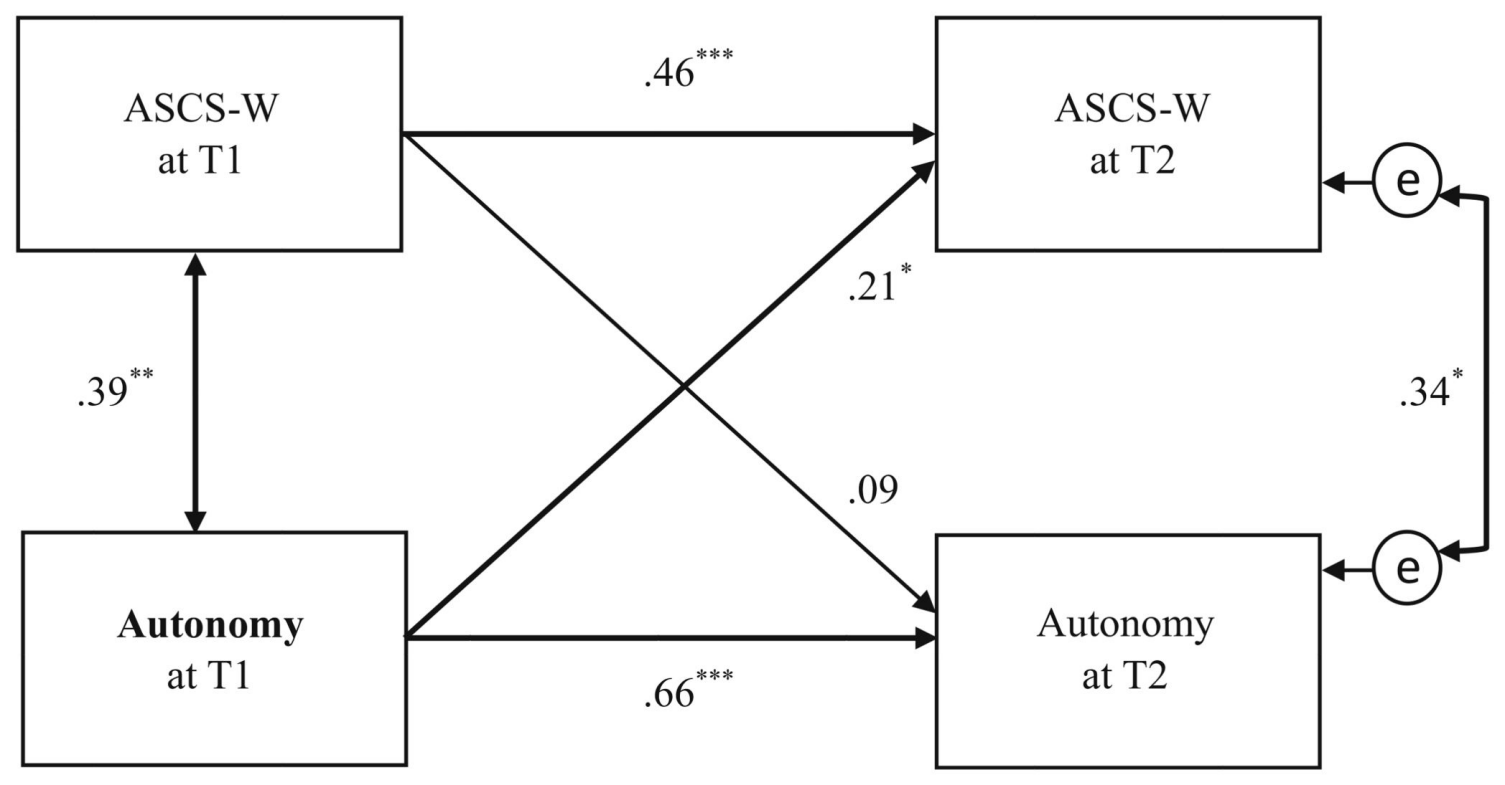
Path model of cross-lagged effects between autonomy and the ASCS-W at T1/T2 (*N* = 72); * *p* ≤ .05, ** *p* ≤ .01, *** *p* ≤ .001

Regarding *autonomy*, the path analyses showed a positive and significant effect (β = .21, *p* ≤ .05) from T1 on ASCS-W at T2, confirming Hypothesis 4a (see Fig. 5). The reverse effect between ASCS-W T1 and *autonomy* T2 was not significant (β = .09, *p* = .35), confirming the hypothesized direction.

In addition, the effect over time (over the 6 months) between the same variable *autonomy* was relatively large (β = .66, *p* < .001) in this model. This indicates a high relation between *autonomy* at T1 and *autonomy* at T2 in the work characteristics of this sample.

**Fig. 6:**
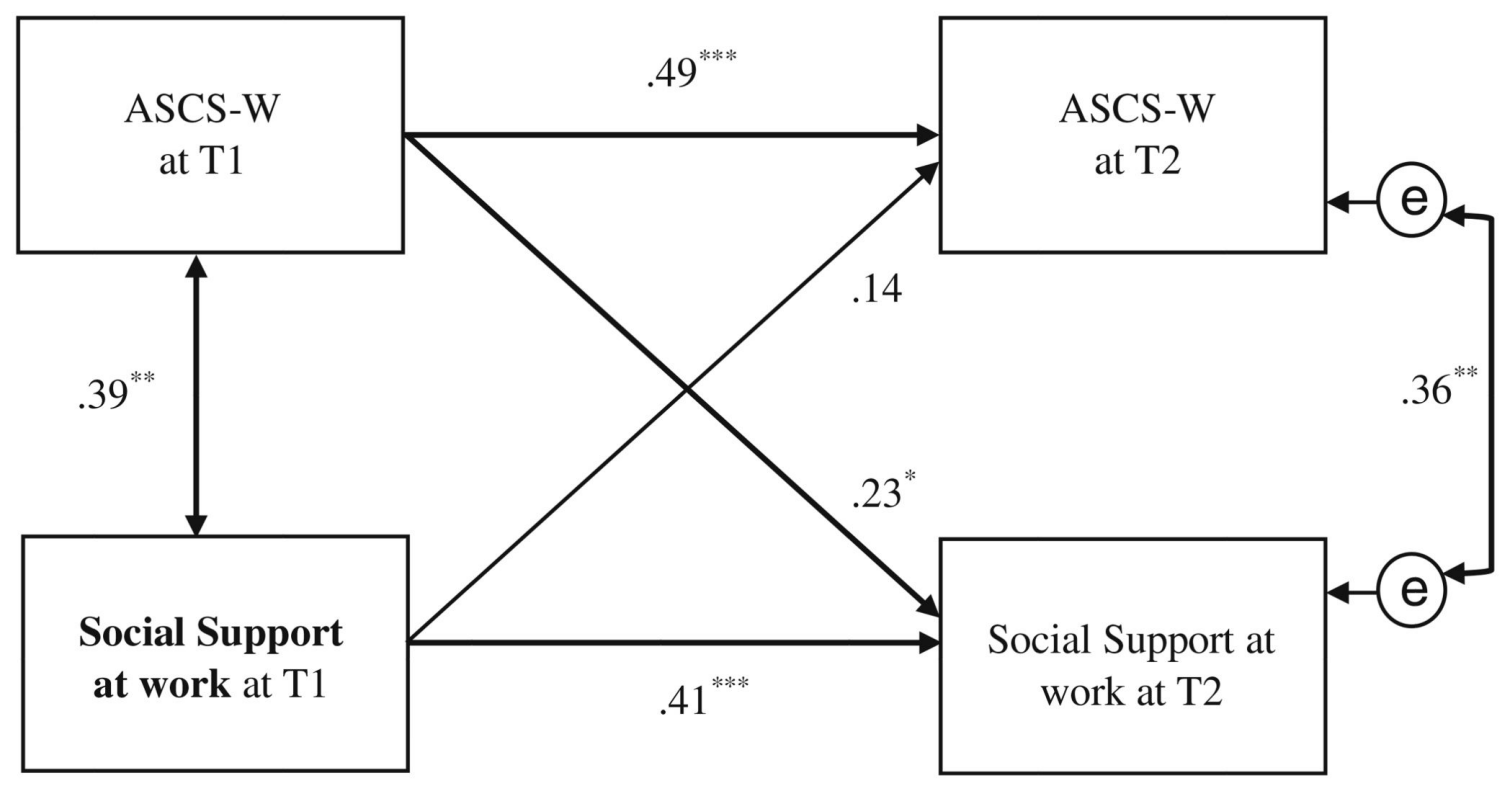
Path model of cross-lagged effects between social support at work and the ASCS-W at T1/T2 (*N* = 72); * *p* ≤ .05, ** *p* ≤ .01, *** *p* ≤ .001

The analyses regarding *social support at work* showed a positive but not significant effect (β = .14, *p* = .21) from T1 on ASCS-W at T2 (see Fig. 6). The reverse effect between ASCS-W T1 and *social support at work* T2 was instead stronger and significant (β = .23, *p* ≤ .05), rejecting the hypothesized *direction* of Hypothesis 4b for *social support at work*.

In addition, the effect between *social support at work* at T1 and *social support at work* at T2 was relatively moderate (β = .41, *p* < .001) in this model, which indicates some changes of *social support at work* over the 6 months in the work characteristics of this sample.

**Fig. 7:**
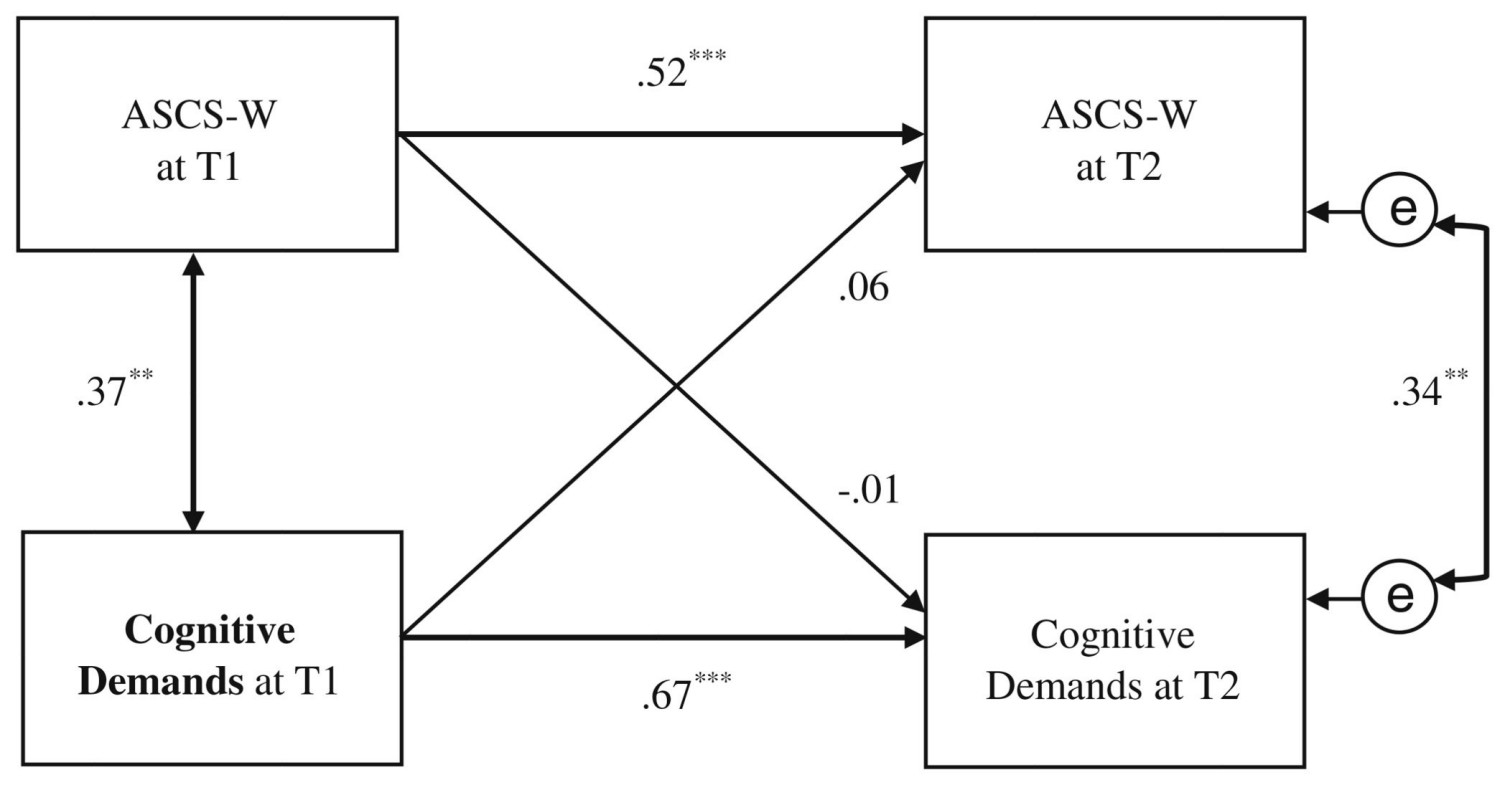
Path model of cross-lagged effects between cognitive demands and the ASCS-W at T1/T2 (*N* = 72); ** *p* ≤ .01, *** *p* ≤ .001

Regarding *cognitive demands*, the analyses revealed neither a significant effect from *cognitive demands* at T1 on ASCS-W at T2 (β = .06, *p* = .58) nor a reversed effect from ASCS-W at T1 to *cognitive demands* at T2 (β = −.01, *p* = .89). Thus, Hypothesis 4c was rejected for this work characteristic (see Fig. 7).

In addition, the relation in the cross-lagged-panel model between *cognitive demands* at T1 and *cognitive demands* at T2 was relatively large (β = .67, *p* < .001) in this sample.

**Fig. 8:**
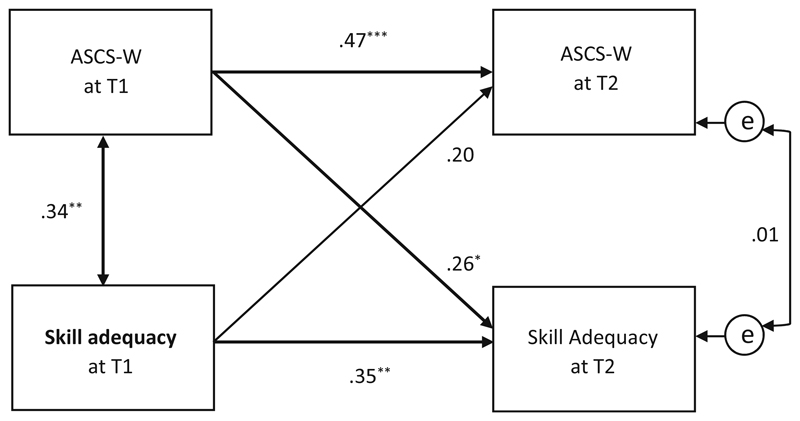
Path model of cross-lagged effects between skill adequacy and the ASCS-W at T1/T2 (*N* = 72); * *p* ≤ .05, ** *p* ≤ .01, *** *p* ≤ .001

The analyses regarding *skill adequacy* showed a positive, insignificant effect (β = .20, *p* = .057) from T1 on ASCS-W at T2 (see Fig. 8). The reversed effect from ASCS-W at T1 to *skill adequacy at* T2 was significant (β = .23, *p* ≤ .05), rejecting the hypothesized *direction* of Hypothesis 4d for *skill adequacy*.

In addition, the effect between *skill adequacy* at T1 and *skill adequacy* at T2 was relatively small (β = .35, *p* < .01), which indicates changes of *skill adequacy* over the 6 months in the work characteristics of this sample.

Finally, the coefficients between ASCS-W at T1 and ASCS-W at T2 ranged between β = .41 and β = .52 in the five cross-lagged panel analyses. These coefficients indicate some changes over the 6 months regarding the applicability of signature character strengths in the work setting of this sample.

## Discussion

The results of our cross-sectional analyses confirmed that all four work characteristics (1) autonomy, (2) social support by supervisors and colleagues, (3) cognitive (challenging) demands and (4) skill adequacy were related positively with the applicability of signature character strengths at work (ASCS-W). Our results also confirmed the relationship between the ASCS-W and work engagement as well as general well-being. The indirect effects from the four work characteristics on work engagement through the ASCS-W and from autonomy, social support and cognitive demands through ASCS-W on general well-being indicate the crucial role of the ASCS-W in terms of workplace motivation and health-promotion.

Hypothesized longitudinal effects between the work characteristics and ASCS-W over a time period of 6 months could be confirmed for autonomy and the combination of all four work characteristics (Fig. 4), but were rejected for the work characteristics of social support at work, cognitive demands and skill adequacy separately. Regarding the picture of the physicians’ work situation, some work characteristics like autonomy and cognitive demands were more strongly related to themselves over time than other work characteristics. Therefore, lower degrees of autonomy and higher degrees of cognitive demands (see also Table 1) seem to be typical work characteristics of hospital physicians (in training) in our sample. Other characteristics, like the ASCS-W, social support at work and particularly skill adequacy changed over the 6 months of the survey-period and seem to be more strongly associated to specific departments and teams. As the main part of the sample were physicians in training, it is not surprising that some characteristics change due to regular rotation of work settings within the training curricula of the medical field. Considering that the changes may have mitigated effects over time, this partially explains some insignificant relations. On the other hand, this circumstance highlights the effect of the significant relation between autonomy and ASCS-W. In other words, while the work characteristics and settings may have (slightly) changed, a significant relational effect between autonomy and ASCS-W still remained over time.

When the results are taken all together, potential *thriving work characteristics* do exist and are related to the applicability of character strengths of hospital physicians at work. According to the cross-sectional analyses, autonomy, social support, cognitive demands, and skill adequacy were shown to be relevant in terms of self-actualization and well-being at work.

Autonomy played a major role in the longitudinal analyses, leading to indications of potential causal relations. The more autonomy a physician has in her/his working tasks, the more room there is for that physician to apply and potentially develop individual strengths beyond the basic requirements. These results support the model of Glaser et al. (2015), the JD-R and Karasek (1979), in which autonomy, as a typical work resource, fosters personal development. As well, results can be related to the Self-Determination-Theory of Ryan and Deci (2000), where autonomy (as a person-related perception) is one of the basic human needs leading to person-coherent behavioral motivation. And last but not least, results match to the concept of job crafting, where people – when they have the possibility (i.e. autonomy) – tend to craft their jobs “by changing cognitive, task, and/or relational boundaries to shape interactions” (p. 179, Wrzesniewski and Dutton 2001). According to Wrzesniewski and Dutton, the individual job crafting can lead to higher work meaning and changes work identity, including different positive work experiences. The application of individual character strengths could just be another aspect, perfectly fitting into this concept.

As we had a majority of young physicians in training in our sample, one aspect should be considered for implications into practice: Enhancing autonomy must not mean letting employees work without guidance, especially in the training phase. Many physicians in training also emphasized this point in personal conversations alongside the survey.

Concerning social support at work, we could not confirm hypothesized effects over time, but found a significant relational effect between the ASCS-W at T1 and social support at T2 in the cross-lagged-panel model. This means that physicians already applying their strengths at work are more likely to receive social support from supervisors and colleagues in the hospital work environment than physicians who are not and would benefit from social support to apply their strengths at work. However, our results still indicate that social support at work and the ASCS-W are connected at the same time. In their diary study, Lavy et al. (2017) *did* find an effect between supervisor support and strengths use over time, but with the different time lag of *one day*. More research is needed to clarify these relations and their circumstances, especially over time.

The work characteristic cognitive demands was related to the ASCS-W at the same time only, as longitudinal cross-lagged analyses did not reveal significant effects between these two variables at all. A possible reason for this result could be the already high level of cognitive demands in our sample of physicians (see Table 1), which did not allow for variance or change over time. Other reasons for this outcome include the inappropriateness of the chosen time lag of 6 months for this specific relationship, no existing longitudinal effects, or the possibility of an interactive effect. According to the Demand-Control Model (Karasek 1979) only the combination of high cognitive demands and high decision latitudes (i.e. autonomy) will result in active learning. As autonomy was comparably low in this study, this combination was unlikely. However, the “effective” combination of all four *thriving work characteristics* on the ASCS-W over time (Fig. 4) may suggest the existence of such interactions. Further studies are recommended to examine possible interactive effects with an adequate sample size.

Similar to results related to social support at work, we also found a reversed significant time-lag effect for ASCS-W impacting the perception of skill adequacy 6 months later. This result could be explained by (1) perceived self-efficacy through ASCS-W in terms of “being at the right (work) place” and (2) the existence of a gain spiral or reciprocal relationship between ASCS-W and skill adequacy based on shared content-related aspects (coherence and adequacy due to P-O fit). In this respect, the hypothesized effect from skill adequacy on ASCS-W was not significant, but fairly strong. Further research is needed to clarify the probability that the missing significance resulted from reduced statistical power due to the small sample size.

The confirmation of ASCS-W affecting work engagement supports existing results concerning the applicability of character strengths and their positive outcomes (e.g., Hausler et al., 2017a, b). Where previous studies focused more on general outcomes (engagement in general, well-being, positive affect etc.; e.g., Harzer and Ruch 2013), we provide evidence of ASCS-W significantly affecting another established work-related well-being aspect. The nonsignificant effect of ASCS-W on general well-being in the indirect-effect model implies weaker effects of ASCS-W on outcomes more distal to the working context. A possible research question to examine in further studies could be why the assumed spillover effect to the physicians’ general / private lives was not great enough. Nevertheless, this result did not support the general positive impact of being able to apply one’s strengths at work on well-being as it was found in other studies (e.g., Hausler et al. 2017a). At the same time, it should be considered that our measure of general well-being is a multidimensional construct where significant effects could exist in some sub dimensions only (e.g., Höge et al. 2018 in this special issue).

The significant indirect effects regarding work engagement and general well-being still revealed the relevant potential of *thriving work characteristics* in combination with the possibility of applying individual character strengths at work in order to promote desirable outcomes like work engagement and general well-being. These results also support potential effect mechanisms through an upward (Fredrickson 2001) or gain spiral (Salanova et al. 2010). Only skill adequacy does not appear to affect general well-being through the ASCS-W in our study.

Limitations of this study concern its small sample size (longitudinal data) and the measurement type of self-assessment survey. Effects that do not result in significance in the context of the longitudinal hypotheses (especially for skill adequacy and potentially also for social support at work) may be explained by the limited sample size given that the coefficients were respectable. A recommended minimal ratio of *cases* to *parameters to estimate* is 10:1 (Kline 2015), implying for example a sample size of *N* = 140 for the path models. Furthermore, self-assessment of work characteristics often raises the question of whether these perceptions fairly and objectively represent working conditions where interventions of work and organizational psychologists would take over. Additional interviews or measures like field observations could provide useful information in this case. Finally, it has to be emphasized that the results of our study may not be representative for either employees or for fully specialized physicians in general, as we primarily examined work characteristics, ASCS-W and work-related well-being of hospital physicians *in training* (89% of the sample). Nevertheless, as Shanafelt and her colleagues (Shanafelt et al. 2015) showed in their comprehensive studies, it is in the first year of training when the risk of experiencing burnout is generally the highest. In addition, our experiences in talking to physicians who took part in the survey were that the last years of specialty training don’t differ much from the work of a specialized physician in the everyday work. Also, the amount of responsibility undoubtedly increases towards the end of the training phase. Thus, the results of this study may also be applicable (at least to a certain degree) to specialized hospital physicians. As the control variable *training status* revealed no relation to well-being outcomes (see Table 1), further studies could also analyze the duration of professional activity (in years) rather than looking at two groups of physicians.

Suggestions for further studies also include the examination of the proposed hypotheses and relations between work characteristics with regard to specific single character strengths or strength-groups (e.g. strengths of the heart/ the mind, strengths related to the self/ others; Niemiec 2012). These strengths (groups) may correspond, more or less, to specific work characteristics: e.g., strengths of mind to cognitive demands or interpersonal strengths to social support. Furthermore, the relations to various outcomes are potentially supposed to vary (e.g., Huber et al. 2018 in this special issue), also considering, for example, the so called “happiness strengths” (e.g., Littman-Ovadia et al. 2017; Hausler et al. 2017b).

### Conclusion and Implications

We identified *thriving work characteristics* that may promote the applicability of individual signature strengths at work (ASCS-W) at specific points in time. In particular job autonomy (i.e. enough latitude for action, decision-making and scope in general) – over time – and the perceived adequacy of one’s own skills for the job, social support at work and cognitive demands – at the same time. Significant indirect effects of the four work characteristics and the ASCS-W on work engagement and of autonomy, social support and cognitive demands on general well-being support the importance of *thriving work characteristics* for physicians’ well-being and health. Regarding the work environment of hospital physicians, we therefore suggest to (a) focus early on the training conditions at medical universities and resource-based curricula to gain subsequent person-job fits and to (b) monitor the trends of restrictions (e.g., overload of bureaucracy, shift work or general work organization) in daily work procedures. These trends of restrictions not only impair motivation and health outcomes, but limit one of the most important *thriving work characteristics* in hospitals: physicians’ autonomy.

## Figures and Tables

**Table 1:** Means, standard deviations, internal consistencies and inter-correlations (T1: standard letters, T2: italicized)

Variable		N	M	SD	α	1		2		3		4		5		6	7	8	9
						T1 | T2		T1 | T2		T1 | T2		T1 | T2		T1 | T2					
Work characteristics																	
1 Autonomy	T1	173	2.55	0.71	.90		.70**												
	*T2*	*66*	*2.44*	*0.82*	.*93*														
2 Social support (colleagues & supervisor)	T1	173	3.31	0.78	.87	.42**	.43**		.51**										
	*T2*	*66*	*3.31*	*0.73*	.*83*	.28*	.44**												
3 Cognitive demands	T1	173	4.22	0.63	.77	.23**	.34**	.23**	.*24*		.67**								
	*T2*	*66*	*4.19*	*0.70*	.*88*	.30*	.43**	.*23*	.32**										
4 Skill adequacy	T1	173	3.52	0.67	.72	.33**	.25*	.30**	.*17*	.22**	.*20*		.45**						
	*T2*	*66*	*3.55*	*0.60*	.*64*	.41**	.51**	.29*	.33**	.*20*	.28*								
Dependent variables																			
5 ASCS-W	T1	173	3.90	0.53	.81	.41**	.38**	.39**	.40**	.38**	.25*	.41**	.40**		.55**				
	*T2*	*69*	*3.76*	*0.57*	.*76*	.40**	.52**	.32**	.52**	.25*	.39**	.37**	.28*						
6 Work engagement	T1	165	3.65	1.12	.94	.25**	.32**	.38**	.35**	.17*	.*24*	.22**	.41**	.43**	.37**				
	*T2*				.*96*														
7 General well-being	T1	160	3.86	0.43	.89	.18*	.29*	.27**	.31*	.20*	.*21*	.19*	.31*	.31**	.43**	.57**			
	*T2*				.*91*														
Control variables																			
8 Gender (1 = female, 2 =male)	T1	173				.05	.*18*	.08	.*004*	.04	.*04*	−.04	.*10*	−.07	.*05*	−.03	−.09		
	*T2*	*72*																	
9 Training status (1 = in training, 2 = specialist)	T1	173				.25**	.38**	.02	−.*11*	.10	.*21*	.29**	.25**	.22**	.*18*	.03	−.02	.11	
	*T2*	*72*																	
10 Age (years)	T1	173	33.4	6.76		.20**	.*17*	−.10	−.*12*	.06	−.*07*	.28**	.26**	.08	−.*03*	−.05	−.002	.01	.55**
	*T2*	*72*	*32.9*	*5.15*															

Data missing if sample sizes do not equal *N* = 173 at T1 or *N* = 72 at T2

*p ≤ .05, **p ≤ .01 (two-tailed)

**Table 2:** Results of the indirect effects on work engagement

Hypothesis	Variables		β	*SE*	*p*	95% *CI*	*R^2^*
H.1	work characteristics - > ASCS-W		Social Support: .19	0.09	≤.05	[.01,.37]	.35
			Autonomy: .20	0.10		[.01,.40]	
			Cognitive Demands: .23	0.07		[.09,.37]	
				0.09		[.02,.39]	
			Skill Adequacy: .21				
H.3a	work characteristics - > ASCS-W - > work engagement	indirect effects	Social Support: .06	0.04	≤.05	[.01,.15] ^*a*^	.25
			Autonomy: .06	0.03		[.01,.14] ^*a*^	
			Cognitive Demands: .07	0.03		[.03,.15] ^*a*^	
				0.04		[.01,.16] ^*a*^	
			Skill Adequacy: .07			

*N* = 165; controlled for gender, age and training status.

a bootstrapping confidence intervals

**Table 3:** Results of the indirect effects on general well-being

Hypothesis	Variables		β	*SE*	*p*	*95% CI*	*R*^2^
H.1	work characteristics - > ASCS-W		Social Support: .18	0.09	.053	[−.002,.36]	.34
			Autonomy: .22	0.10	≤.05	[.01,.42]	
			Cognitive Demands: .23	0.07	≤.05	[.09,.37]	
				0.09	≤.05	[.01,.38]	
			Skill Adequacy: .20			
H.3b	work characteristics - > ASCS-W - > general well--being	indirect effects	Social Support: .04	0.03	≤.05	[.0001,.12] ^*a*^	.15
			Autonomy: .04	0.03	≤.05	[.001,.12] ^*a*^	
			Cognitive Demands: .05	0.03	≤.05	[.005,.11] ^*a*^	
				0.03	≥.05	[.000,.12] ^*a*^	
			Skill Adequacy: .04			

*N* = 160; controlled for gender, age and training status.

a bootstrapping confidence intervals
